# Design and Fabrication of Polymer Triboelectric Nanogenerators for Self-Powered Insole Applications

**DOI:** 10.3390/polym15204035

**Published:** 2023-10-10

**Authors:** You-Jun Huang, Chen-Kuei Chung

**Affiliations:** Department of Mechanical Engineering, National Cheng Kung University, Tainan 701, Taiwan

**Keywords:** mechanical energy harvesters, triboelectric nanogenerators, polydimethylsiloxane, human–machine interface, polymer

## Abstract

Triboelectric nanogenerators (TENGs) are a kind of mechanical energy harvester with a larger force sensing range and good energy conversion, which is often applied to human kinetic energy collection and motion sensing devices. Polymer materials are the most commonly used materials in TENGs’ triboelectric layers due to their high plasticity and good performance. Regarding the application of TENGs in insoles, research has often used brittle Teflon for high output performance together with hard materials, such as springs, for the mechanism to maintain its stability. However, these combined materials increase the weight and hardness of the insoles. Here, we propose a polyethylene terephthalate (PET)-based TENG with a micro-needle polydimethylsiloxane (PDMS) elastomer, referred to as MN-PDMS-TENG, to enhance performance and maintain comfort flexibility, and structural stability. Compared with a flat PDMS, the TENG with a microstructure enhances the output open-circuit voltage (Voc) from 54.6 V to 129.2 V, short-circuit current (Isc) from 26.16 μA to 64.00 μA, power from 684 µW to 4.1 mW, and ability to light up from 70 to 120 LEDs. A special three-layer TENG insole mechanism fabricated with the MN-PDMS-TENG and elastic materials gives the TENG insole high stability and the ability to maintain sufficient flexibility to fit in a shoe. The three-layer TENG insole transforms human stepping force into electric energy of 87.2 V, which is used as a self-powered force sensor. Moreover, with the calibration curve between voltage and force, it has a sensitivity of 0.07734 V/N with a coefficient of determination of R^2^ = 0.91 and the function between force and output voltage is derived as F = 12.93 V − 92.10 under human stepping force (300~550 N). Combined with a micro-control unit (MCU), the three-layer TENG insole distinguishes the user’s motion force at different parts of the foot and triggers a corresponding device, which can potentially be applied in sports and on rehabilitation fields to record information or prevent injury.

## 1. Introduction

Triboelectric nanogenerators (TENGs) are a kind of mechanical energy harvester, used to convert mechanical energy into electrical energy or signals through the interactive motion between two triboelectric layers. Such motion includes vertical motion and parallel motion. These two forms of movement can be further divided into four forms through the combination of electrodes, namely, vertical contact–separation mode, lateral sliding mode, single-electrode mode, and freestanding triboelectric-layer mode [1]. Most of the kinetic energy existing in daily life can be converted by these four modes. For example, wind energy can be converted by the free-standing mode [2], and the energy of waves can be converted by the vertical contact–separation mode [3] or free-standing mode [4], depending on the mechanism. To a great degree, the majority of human body movement is vertical movement and can trigger the vertical contact–separation mode and single-electrode mode of TENGs. Some examples of this vertical body movement are pressing a keyboard [5,6], walking on carpet [7], and stepping on insoles [8]. Compared with other energy harvesters such as piezoelectric nanogenerators (PENGs) and electromagnetic generators (EMGs), TENGs capture human motion energy in a higher force range, which means that TENGS have greater potentiality to be applied to harvesting human motion.

Conventional TENGs are made by combining two triboelectric layers made of dielectric materials or one metal material layer combined with another dielectric layer. The material properties of these two triboelectric layers will critically affect the output electrical performance, mechanical properties, and application range of the TENG [9]. When the two triboelectric layers move relative to each other, electrons are exchanged between the two triboelectric layers, thereby generating triboelectricity. The quantity of electrons involved in the movement and the tendency of transfer depend on the triboelectric series of the two triboelectric layers. The greater the triboelectric series difference, the stronger the electron transfer tendency is, and the greater the open-circuit voltage and short-circuit current will be. Therefore, finding an appropriate combination of materials with suitable mechanical properties and a sufficiently high triboelectric series difference is crucial for TENG applications. As a dielectric material, compared with metal materials and oxide materials, polymer materials such as polydimethylsiloxane (PDMS) [10] and rubber [11] have the potential to maintain a high triboelectric series difference between triboelectric materials while maintaining elasticity and plasticity. Due to the higher positive tribo-polarity, people tend to use polymer Teflon to synthesize the dielectric layer of the TENG [12,13]. However, the elasticity of Teflon after curing is poor compared with other polymer materials used in TENGs. Such materials may have problems of durability and discomfort when used for human motion sensing. Therefore, using elastic PDMS to achieve a sufficiently high electrical output performance is a key challenge for TENG human motion sensors.

The direction of energy received by the insole from human motion is mostly vertical contact and separation; insoles are a major application field of vertical contact–separation TENGs in human body sensing. To maintain the shape and stability of the TENG in insoles, many studies have used a two-layer mechanism in TENG insoles and added spacers such as a spring [10]. However, such mechanisms have a very high rigidity, volume, and weight [8,14], which not only causes great discomfort to the user, but also reduces the number of TENGs that can be placed in one insole. This greatly reduces the scalability of the TENG insole force sensor. Therefore, it is very important to find a flexible, thin, and light TENG insole mechanism. TENGs produce different open-circuit voltages under different forces; therefore, researchers have analyzed the relationship between the open-circuit voltage and the force applied to a TENG to determine the sensitivity of the TENG and use TENGs as force sensors [15]. However, most of the research on TENG insoles applied as force sensors only covers tiny forces between 1 and 40 N [16,17,18], which is less than the actual applied force on an insole. However, TENGs have the characteristic of having different force sensitivities in different force ranges. Generally, the higher the force range measured, the lower the force sensitivity of the TENG [19,20]. Determining the force sensitivity of the TENG in the force range of an insole absorbing energy while the user is walking and running is necessary for the TENG insole force sensor. However, the force sensitivity measurement of a TENG in large force ranges is very rarely undertaken; thus, determining the force sensitivity of a TENG in the human pedaling force range is a necessary step for making TENG insole force sensors.

The combination of TENGs and a microcontroller unit (MCU), such as an Arduino UNO, enables the electrical signals converted from mechanical signals by TENGs to be directly applied to control low-power-consuming devices [21,22]. In addition to force sensitivity and output electrical performance, size, thickness, and comfort must be taken into consideration [21,23]. The choice of materials for the triboelectric layers in a TENG insole and the selection of the spacer material will significantly impact its usability.

Here, we propose a TENG with a micro-structured PDMS to enhance the output performance, using polyethylene terephthalate (PET) as a base material to maintain its comfort, flexibility, and structural stability. The other triboelectric layer of the TENG is the conductive layer, which uses a conductive cloth with a nylon cloth base (nylon cloth based conductive cloth, NBCC). All materials of this TENG are composed of elastic, flexible, and lightweight soft materials, unlike traditional TENG insoles which use hard and brittle material [11,24]. The microstructures on the surface of the PDMS dielectric layer are generated by laser processing on a PMMA mold and demolding. The microstructures can provide a higher effective contact area under the premise of the same test piece area, allowing more electron charging and discharging during movement: the Voc of the TENG with microstructure increased from 54.6 V to 129.2 V; the Isc increased from 26.16 μA to 64.00 μA; the power increased from 684 μW to 4.1 mW; and the number of lighting LEDs increased from 70 to 120. Through the microstructure, PDMS-TENGs can have a sufficiently high electrical signal to be applied in TENG insoles. In addition, we propose a three-layer insole mechanism that uses elastic PE as a spacer, which ensures that each stepping and releasing of the user completely triggers a contact and separation movement, which generates an electrical signal corresponding to the user’s stepping force. There are no brittle materials; therefore, the three-layer TENG insole mechanism avoids obvious material damage after long-term wear as an insole by the user. Through the data analysis and sorting between the output voltage of MN-PDMS-TENG insoles and forces applied, the calibration curve is built in the sensitivity of 0.07734 V/N with the coefficient of determination R^2^ = 0.91 in the range of human stepping forces (300~550 N). With the formula of the calibration curve, F = 12.93 V − 92.10, the stepping forces of the user’s different parts, such as the forefoot and rear foot, are measured through the output voltage and used to determine the user’s state from walking, running, and standing.

The system combines a three-layer TENG insole with an Arduino UNO to sense the stepping state of a human. This system senses the force distribution of the user’s feet, converts it into an electronic signal, and outputs corresponding information according to the user’s state in a very low electric power consumption. For some sports and rehabilitation, athlete and patient heart rates need to be controlled within a certain range, but full-time monitors require lots of electrical power support. Three-layer TENG insoles reduce the excess use of electrical power while being used as the self-powered force sensor that prevents heart injury. With more application tests in the future, three-layer TENG insoles can analyze the user’s stepping force distribution to help rehabilitation.

## 2. Materials and Experimental Methods

### 2.1. The Material Selection and Fabrication of PDMS-TENGs

To make elastic and soft TENG insoles, we chose PDMS as the dielectric layer material. PDMS is a flexible and stretchable silicone material. As a dielectric material, however, it cannot provide a high enough triboelectric series distance between metal materials, like Teflon. However, PDMS has extremely high plasticity before curing, and various microstructures, such as microneedles [25] and microparticles [26], can be produced through molding and over-molding to increase the effective contact area. Therefore, compared with other materials used as a TENG’s dielectric layer, we can form a variety of microstructures on the surface of the PDMS dielectric layer to improve its electronic conversion tendency during a load, and further improve its electrical output performance. In this study, PDMS, mixed with an elastic agent and curing agent at a ratio of 1:10, was used as the dielectric layer material of TENG. Therefore, the microneedle structure formed by CO_2_ LASER involving a poly methyl methacrylate (PMMA) mold was used to enhance the electrical performance of PDMS-TENG.

The vertical pressure and lateral friction exerted by humans on the insole during walking are huge for TENGs. Even though PDMS is an elastic material, when it is placed in the insole for a long time, the forces between the shoes and feet will deform the PDMS dielectric layer, thereby reducing the output performance and force sensitivity of the PDMS TENG insole. We utilized the property of PDMS, which allows it to penetrate a material before curing and then coat the material after curing, to facilitate the penetration and coating of PET and conductive cloth [24]. In this way, PET became the fiber-based PDMS-TENG to withstand the excessive external force to protect the PDMS-TENG from being damaged. Destruction can also stabilize the shape of the TENG insole, stabilizing its stable electrical output properties and force sensitivity.

**Figure 1:**
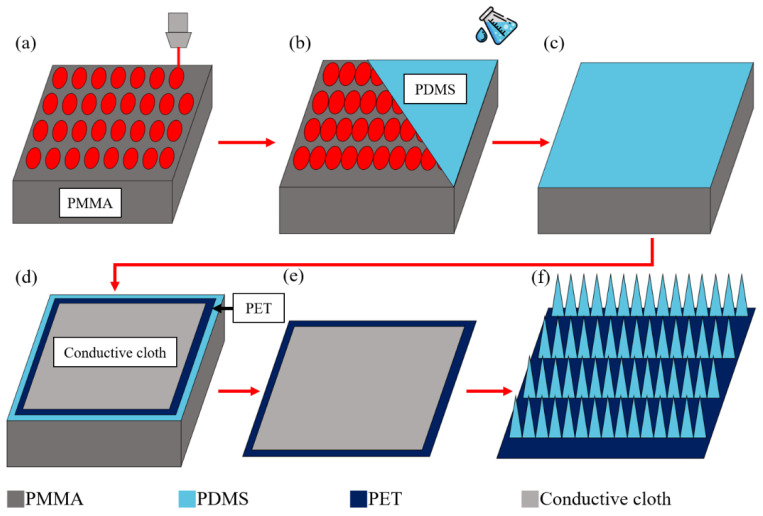
Process of MN-PDMS-TENG synthesis: (**a**) microneedle arrays are engraved on 6 × 6 cm^2^ PMMA mold by a CO_2_ laser; (**b**) PDMS is perfused onto PMMA to fill the microneedle array; (**c**) the PDMS and PMMA mold are put into a vacuum chamber of 100 mTorr to remove the bubbles from the structure; (**d**) the PET and conductive cloth are placed onto the PDMS and put into a vacuum chamber of 100 mTorr to remove the air inside the fiber and coat the fiber with PDMS; (**e**) the sample is heated in an oven at 80 degrees Celsius for one hour and the MN-PDMS-TENG is demolded; (**f**) after cooling the MN-PDMS-TENG naturally, the MN-PDMS-TENG can be used.

To synthesize the fiber-based microneedle PDMS dielectric layer, we first used a CO_2_ laser to process a microneedle array(5 × 5 cm^2^) with a specified depth on a PMMA mold (6 × 6 cm^2^), as shown in Figure 1a. Secondly, we filled the mold with a PDMS silicone liquid mixed with an elastic agent and curing agent at a ratio of 1:10, as shown in Figure 1b, and placed the sample into a vacuum chamber with 100 mTorr to remove air bubbles inside the microneedle structure for 10 min, as shown in Figure 1c. Then, the conductive cloth and PET were placed on the PDMS, and the sample was put into the vacuum chamber of 100 mTorr for 30 min to allow the fibers to completely sink into the PDMS, as shown in Figure 1d. The sample was then heated in an oven at 80 degrees Celsius for one hour to cure completely and then released from the mold to obtain the PDMS-TENG with the microneedle (MN-PDMS-TENG), as shown in Figure 1e. After the MN-PDMS-TENG had cooled to room temperature naturally, the MN-PDMS-TENG could be used in the test shown in Figure 1f.

### 2.2. Experiment and Measurement of MN-PDMS-TENG

The conductive layer of TENG is primarily composed of metal materials such as aluminum and copper. Nevertheless, incorporating a metal material layer into the insole can result in an excessive strain storage capacity, requiring the user to exert more force while stepping on it compared with a regular insole. For ordinary users, this is particularly uncomfortable; therefore, we used conductive cloths made of nickel-plated cotton cloth as the conductive layer of the TENG. Conductive cloth loses the elasticity of the cotton fiber due to electroless plating and becomes exceptionally fragile. It can easily break during movement when it is used as the TENG’s conductive layer. Therefore, a layer of nylon is pasted on the backside of the conductive cloth to make a nylon base conductive cloth (NBCC). The nylon base absorbs the excessive stress of the conductive cloth during the contact and separation load to prevent the conductive cloth from breaking. Connecting the MN-PDMS-TENG to NBCC forms a vertical contact–separation mode TENG, and was used in the subsequent test.

### 2.3. The Experiment and Measurement of PDMS-TENG and PDMS-TENG Insole

**Figure 2:**
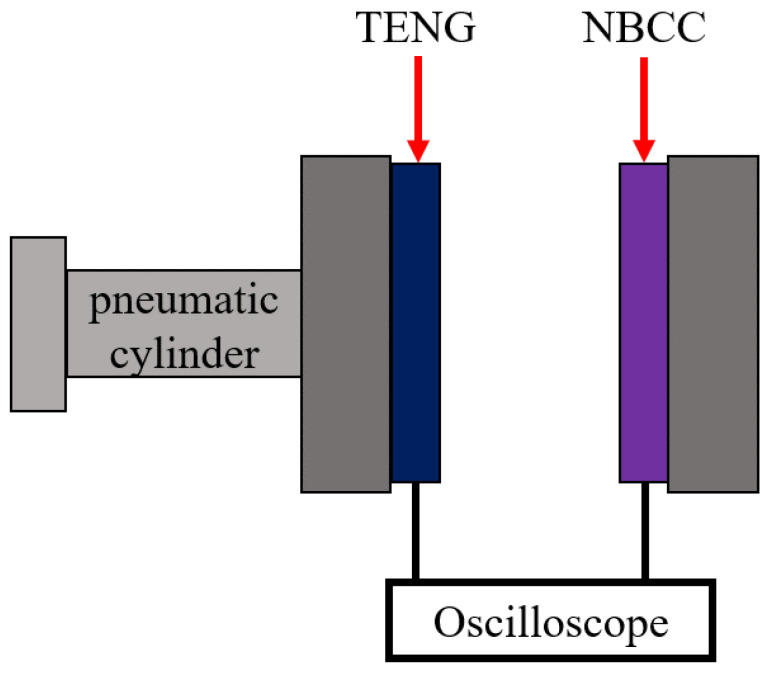
The combination of the pneumatic cylinder actuation platform with the PDMS-TENG and NBCC. The PDMS-TENG was fixed on the shaft of the pneumatic cylinder, and NBCC was fixed on the solid end. When the pneumatic cylinder started working, these two ends initiated the contact and separation movement.

The electrical output performance of the MN-PDMS-TENG was evaluated using a pneumatic cylinder (FESTO D: S-PAZ-DW20-100PPV, Esslingen, Germany) actuation platform. This platform operates in a vertical contact and separation mode, generating steady high-frequency motion to maximize the MN-PDMS-TENG’s output. The MN-PDMS-TENG was fixed onto the shaft of the pneumatic cylinder through the PMMA plate, and NBCC was fixed on the other end, as shown in Figure 2. The open-circuit voltage (Voc) could be measured by connecting the two layers to an oscilloscope (HIOKI Memory HiCorder MR8870-20, Nagano, Japan). The two layers were connected to the circuit; thus, we could obtain the short-circuit current (Isc), output power, the ability to charge capacitors, and the number of LEDs lit up could be measured.

### 2.4. The Design and Process of the Three-Layer TENG Insole

**Figure 3:**
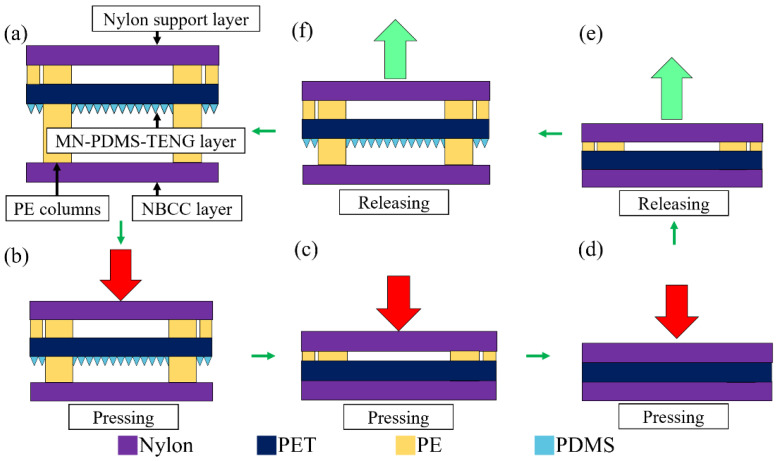
The contact and separation of the MN-PDMS-TENG inside the three-layer TENG insole mechanism: (**a**) the initial state of the three motion system; (**b**) when the three motion system receives the vertical stepping force, the PDMS-TENG layer and NBCC layer start getting closer; (**c**) the PDMS-TENG layer and NBCC layer are in complete contact and the nylon support layer gets close to the back side of PDMS-TENG; (**d**) the nylon support layer and PDMS-TENG are fully in contact; (**e**) when the force releases, the nylon support layer first separates from the PDMS-TENG; (**f**) the NBCC layer separates from the PDMS-TENG layer after the nylon support layer completely separates from PDMS-TENG layer and restores the three motion system to the initial state.

In order to make MN-PDMS-TENG into an insole that could perform stable contact and separation movements with the user stepping, we needed to place the spacers between the MN-PDMS-TENG and NBCC to maintain the separation distance between two triboelectric layers. However, no matter what kind of material is compressed, it will still have a certain thickness, which will affect the deformation of the microneedle structure of MN-PDMS-TENG and prevent the complete contact and separation movement of MN-PDMS-TENG. Therefore, we designed a unique three-layer TENG insole mechanism composed of these three layers: nylon support layer, MN-PDMS-TENG layer, and NBCC layer. Eight polyethylene (PE) columns were used as spacers in the three-motion system to stabilize the shape of the insole, as shown in Figure 3a. The nylon support layer was a 6 × 6 cm^2^ piece of nylon fiber. When the three-layer TENG insole mechanism receives vertical stepping force, the lower strain makes the MN-PDMS-TENG layer come into contact with the NBCC layer first, shown as in Figure 3b,c. After the MN-PDMS-TENG layer is in complete contact with the NBCC layer, the nylon support layer will make contact with the back side of the PDMS-TENG, as shown in Figure 3c,d. When the stepping force releases, the nylon support layer first separates from the PDMS-TENG layer because the strain between these two layers is large, as shown in Figure 3d,e. After the nylon support layer and PDMS-TENG layer are completely separated, the MN-PDMS-TENG layer starts to separate from the NBCC layer, as shown in Figure 3e,f. After the contact and separation between the three layers is complete, the three-layer TENG insole mechanism is fully restored and is ready to take the next stepping force, as shown in Figure 3a–f. This three-layer TENG insole mechanism ensures that the microneedle structure of the MN-PDMS-TENG is completely compressed, deformed, and restored with each step, thereby ensuring that each step makes full contact and separation. As long as the stepping force is continuously applied and released, the three-layer TENG insole mechanism continues working and releases electrical signals with energy from the MN-PDMS-TENG.

In order to insert the PE columns into the three-layer TENG insole mechanism, we cut four 1 × 1 cm^2^ square holes into the MN-PDMS-TENG layer, put four high PE columns connecting the nylon support layer and NBCC layer, and sewed PE columns with two layers with cotton thread. Four low PE columns connected the nylon support layer and NBCC layer, sewed in the same way. To measure the distance between the layers that was most suitable for the three-layer TENG insole mechanism, we used a stepping motor (TOYO CGTY5-L5-100-M-TC100-03, Tainan, Taiwan) actuation platform to measure the output voltage of the MN-PDMS-TENG under various strokes distance, from 4 mm to 10 mm, and selected the most suitable thickness for the insole. The stepper motor enabled precise adjustment of the working stroke, enabling stable reciprocating motion. This capability allowed for the precise fine-tuning of the spacer thickness in the three-layer TENG insole.

**Figure 4:**
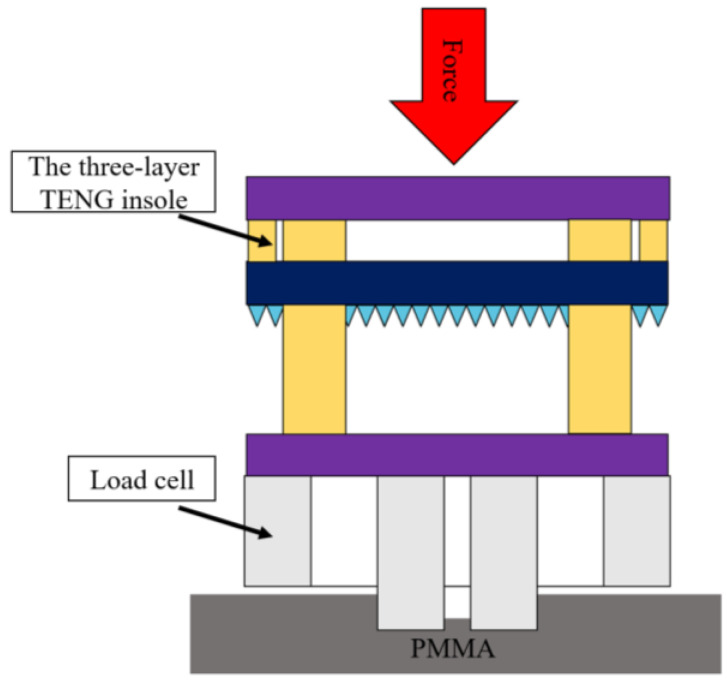
The force measuring system of the three-layer TENG insole. The load cell was placed under the insole to measure the force applied on the three-layer TENG insole. The PMMA was used to absorb the horizontal force and stabilize the load cell. With the data of voltage and force under different forces, the force sensitivity of the three-layer TENG insole could be calculated.

In order to measure the force sensitivity of the three-layer TENG insole, we asked different users to step on the three-layer TENG insole with different forces (300~550 N), similar to the force that acts on the forefoot and heel of the feet when walking and running. As shown in Figure 4, we placed a load cell under the PDMS-TENG insole and fixed it on PMMA to ensure that there would be no slippage when stepping on it. To determine the force sensitivity of the PDMS-TENG within the range of 300 to 550 N, we measure the Voc at various force levels using an oscilloscope. By analyzing the relationship between force and voltage, we could obtain the desired sensitivity data.

## 3. Results and Discussion

### 3.1. The Electric Output Performance of MN-PDMS-TENG

**Figure 5:**
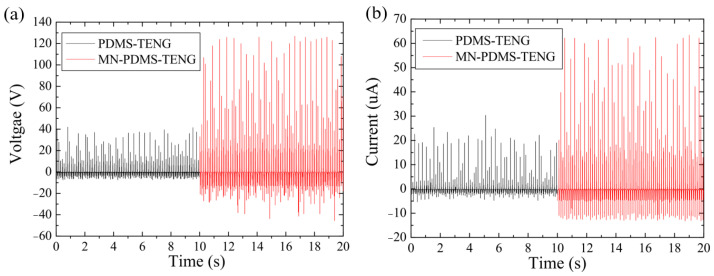
Electrical output performance of the PDMS-TENG and MN-PDMS TENG: (**a**) Voc values of the PDMS-TENG and MN-PDMS-TENG are 54.6 V and 129.2 V, respectively; (**b**) Isc values of the PDMS-TENG and MN-PDMS-TENG are 26.16 μA and 64.00 μA, respectively.

When PDMS is employed as a dielectric layer in TENGs, an increase in the effective contact area of the PDMS dielectric layer results in a greater exchange of charges during triboelectric motion. The microneedle structure can provide a more effective contact area for the PDMS-TENG, thereby improving its electrical performance. By measuring the Voc and Isc of MN-PDMS-TENG and PDMS-TENG without the microneedle structure through the oscilloscope on the pneumatic cylinder actuation platform with a frequency of 8 Hz and an applied force of 16 N, we determined that the Voc of PDMS-TENG without the microneedle structure was 54.6 V, and the Isc was 26.16 μA. The Voc of the MN-PDMS-TENG was 129.2 V, and the Isc was 64.00 μA, which had increased by 237% and 245% compared with the PDMS-TENG without microneedle structure, respectively, as shown in Figure 5a,b. Compared with other TENGs used as insoles, it has relatively high output voltage, current, and power, as shown in Table 1. The higher output voltage and current means that this TENG insole can be applied more easily in force sensing. The power output of the PDMS-TENG and MN-PDMS-TENG under load was calculated through the formula of W = I^2^R. We measured the output power of the PDMS-TENG and MN-PDMS-TENG, which were found to be 684 µW and 4.1 mW, respectively, both under a 1 MΩ load. The MN-PDMS-TENG increased its output power by 599% due to the microneedle structure. Compared with our previous research [25], the Voc and Isc increased by 175% and 178%, respectively, and the power also increased by 316%, as listed in Table 1. The enhanced electric output performance resulted from a higher laser processing power, larger microneedle height, and more total effective contact area. Better electrical output performance means that TENGs can drive more electrical appliances with higher voltage requirements, which also makes the MN-PDMS-TENG more suitable for insoles than those presented in previous studies.

**Figure 6:**
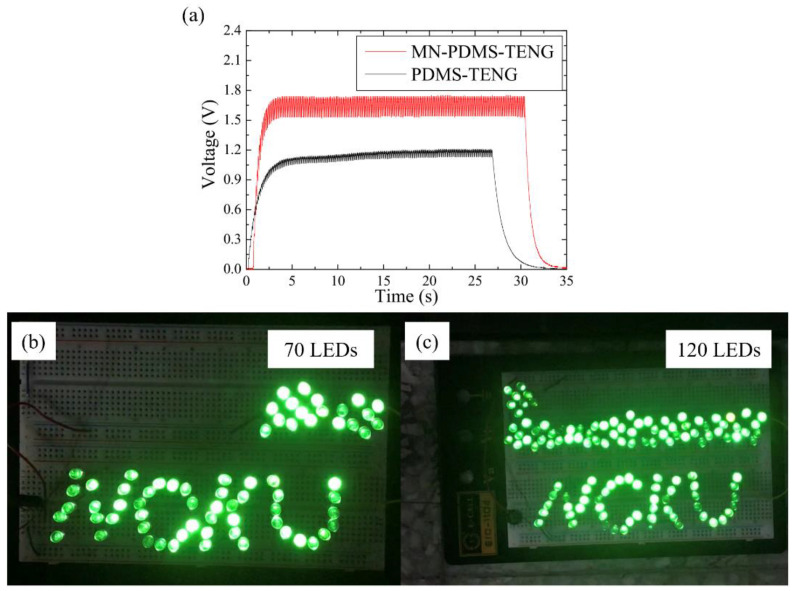
The 1 μF capacitor charging ability of the PDMS-TENG and MN-PDMS TENG: (**a**) the PMDS-TENG reached 1.21 V in 4 s; the MN-PDMS-TENG reached 1.75 V in 3 s; (**b**) 70 LEDs were lit by the PDMS-TENG; and (**c**) 120 LEDs were lit by the MN-PDMS-TENG.

The signals of TENGs mostly fluctuate with movement; therefore, they need to be stored in a capacitor and converted into a stable voltage power supply to drive electronic devices. A TENG with a higher voltage output can charge a capacitor more effectively, enabling it to power a greater number of electronic devices. In order to determine whether the PDMS-TENG and MN-PDMS-TENG had the potential to drive electronic appliances, they were connected to a bridge rectifier and a 1 μF capacitor connected in parallel with a 1 M Ω resistor and switched off through a switch to disconnect the capacitor from the circuit. After turning the switch on, the capacitor began to charge through the PDMS-TENG or MN-PDMS-TENG. When the voltage of the capacitor was stable, we turned the switch off to simulate the process of PDMS-TENG charging and discharging from the capacitor to the electrical application during motion. The PDMS-TENG took 4 s to charge to 1.21 V. The MN-PDMS-TENG took 3 s to charge to 1.75 V, as shown in Figure 6. Under the same capacitance, a higher voltage means that more elements can be driven to operate, which also means that the microneedle structure can improve the application potential of PDMS-TENG.

To evaluate the capacity of both the PDMS-TENG and MN-PDMS-TENG in powering electronic devices, we conducted LED lighting tests as a benchmark. The PDMS-TENG and MN-PDMS-TENG were connected to bridge rectifiers and LEDs, respectively. The PDMS-TENG could light up to 70 LEDs, as shown in Figure 6b. The MN-PDMS-TENG could light up to 120 LEDs, as shown in Figure 6c. The microneedle structure increased the LED’s light-up ability of PDMS-TENG by 171%; this is higher than most TENG insoles, as shown in Table 1. This implies that the MN-PDMS-TENG exhibits superior capability in powering electronic devices compared with the majority of TENG insoles.

### 3.2. The Electric Output Performance of the Three-Layer TENG Insole

In order to set the thickness of spacers inside the three-layer TENG insole with the best power generation efficiency, we needed to measure the electrical output performance of the MN-PDMS-TENG under different strokes of performing the contact and separation motion to determine the optimum distance between the MN-PDMS-TENG layer and the NBCC layer.

**Figure 7:**
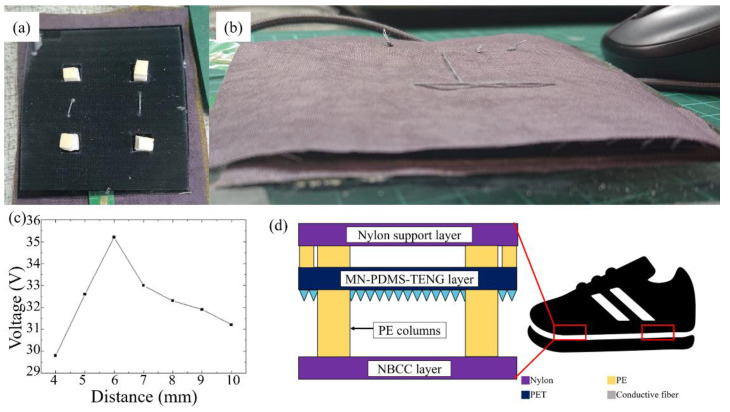
The thickness setting of the three-layer TENG insole: (**a**) the spacers between the NBCC layer and the nylon support layer placed inside the MN-PDMS-TENG layer; (**b**) the three-layer TENG insole after being sewn with the cotton thread; (**c**) the Voc of the MN-PDMS-TENG under different strokes (4~10 mm), with the maximum voltage (35 V) occurring with a stroke is 6 mm; (**d**) the PDMS-TENG insole placed on the forefoot and rear foot inside the shoe.

Here, we placed the MN-PDMS-TENG and NBCC on the different ends of a stepping motor actuation platform and measured the Voc generated when the MN-PDMS-TENG performs came into contact and separated with a stroke of 4~10 mm. The MN-PDMS-TENG generated a maximum voltage of 35 V with 6 mm strokes within other distances, as shown in Figure 7c. Adjusting the thickness of spacers between the nylon support layer and NBCC layer of the three-layer TENG insole to 10 mm and the spacers between the nylon support layer and PDMS layer of PDMS-TENG insole to 4 mm, the three-layer TENG insole was fabricated with the stroke between two triboelectric layers as 6 mm, as shown in Figure 7a,b. Placing the three-layer TENG insole on the forefoot and rear foot inside the shoe indicate its use as a self-powered force sensor, as shown in Figure 7d.

**Figure 8:**
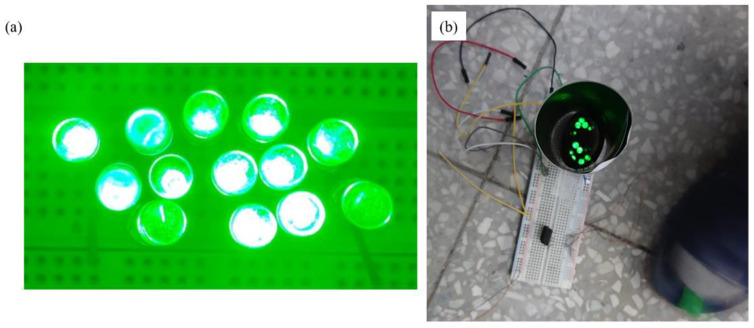
(**a**,**b**) The three-layer TENG insole lighting up 13 LEDs with the stepping force of a person’s forefoot. The three-layer TENG insole inside the forefoot of the slipper is connected to the circuit of the LEDs and a bridge rectifier.

To confirm that the MN-PDMS-TENG maintained its electrical output performance within the three-layer mechanism, we connected the three-layer TENG insole to the circuit, including LEDs, and placed it inside a slipper. A person then stepped onto it using their forefoot. The three-layer TENG insole lit up 13 LEDs, as shown in Figure 8b. By integrating the circuit and LEDs into the footwear, it could be repurposed into a warning light for safer night-time walking and running. 

### 3.3. Human Walking and Running Force Detection

**Figure 9:**
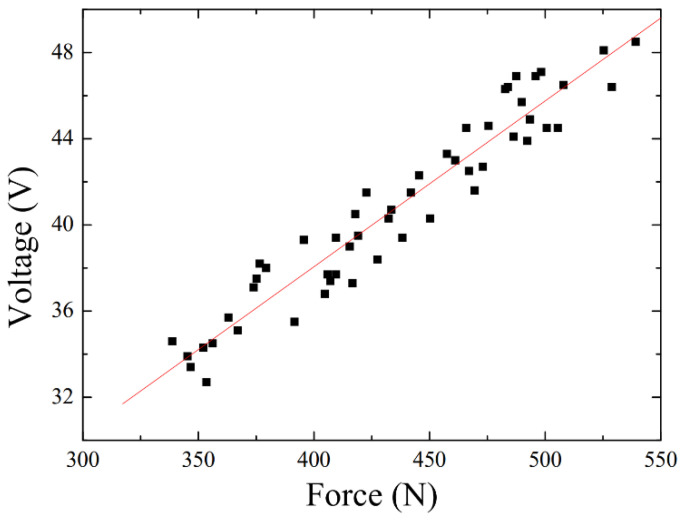
The force sensitivity of the three-layer TENG insole between 300 N and 550 N; the sensitivity of the three-layer TENG insole was 0.07734 V/N with the coefficient of determination: R2 = 0.91. The formula of the sensitivity of the three-layer TENG insole is derived as F = 12.93 V − 92.10.

We put the three-layer TENG insole onto the load cell and fixed the load cell on the PMMA platform. Due to the vertical pressures of each full section in the same system being equal, the vertical force depending on the three-layer TENG insole is equal to the force measured by the load cell. The horizontal force does not affect the force that the load cell measures and the voltage that MN-PDMS-TENG performs; however, the major horizontal force is adsorbed by the PMMA platform to prevent sliding during testing. By collecting data of the force signal measured by the load cell and the voltage signal measured by the TENG insole when the user walked on the load cell and the three-layer TENG insole, a graph of the relationship between voltage and force was built in the range of force between 300 N and 550 N. Here, the positive open-circuit maximum voltage was used as a comparison because the differences in positive voltage were more stable than the negative voltages under forces between 300 N and 550 N. After building the calibration curve, the force sensitivity of the three-layer TENG insole between 300 N and 550 N was obtained as 0.07734 V/N, R^2^ = 0.91, and the function between force and voltage was F = 12.93 V − 92.10, as shown in Figure 9, which means that the three-layer TENG insole can clearly and steadily calculate the user’s stepping force by measuring the voltage produced by the MN-PDMS-TENG when the three-layer TENG insole is put into the user’s shoes. The TENG exhibited different force sensitivities in different ranges [29]. Generally, TENGs have a lower force sensitivity in a higher force range; thus, this result is reasonable. Compared with most of the TENG insole research, which can only distinguish the force of different intervals in a small force range (0 N~300 N), the three-layer TENG insole can not only be used in the large force range (300 N~550 N), but can also calculate the user’s stepping force through the calibration curve function, F = 12.93 V − 92.10, as shown in Table 2.

### 3.4. Human Stepping State Sensing System with the Three-Layer TENG Insole

**Figure 10:**
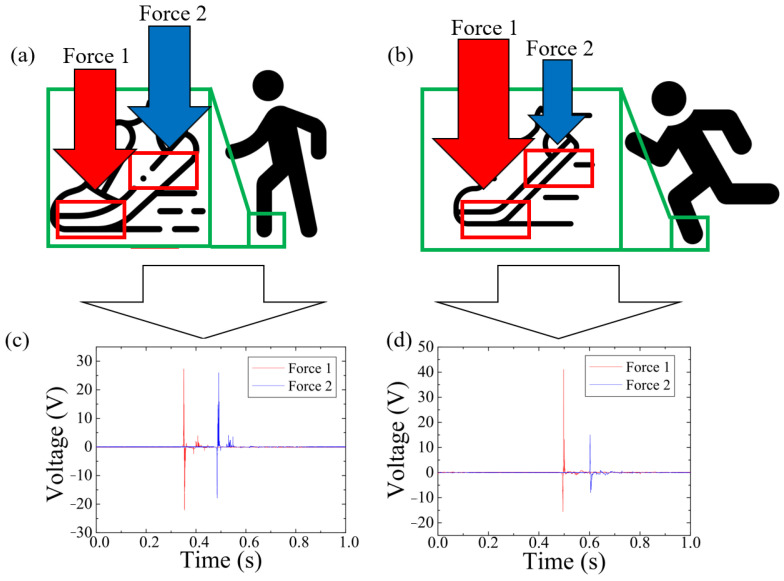
Stepping force sensing of the three-layer TENG insole. (**a**,**c**) While the user was walking, the voltage output of the three-layer TENG insole inside the forefoot part of insole (Force 1) was 28 V and that inside the rear foot part (Force 2) was 26 V. These data were converted into 269.94 N applied on the forefoot and 244.08 N applied on the rear foot. (**b**,**d**) While the user was running, the voltage output of the three-layer TENG insole inside the forefoot part of the insole (Force 1) was 45 V and that inside the rear foot part (Force 2) was 15 V. These data were converted into 489.75 N applied on the forefoot and 101.85 N applied on the rear foot.

Some people are used to landing on the forefoot first while running; therefore, the force applied on the forefoot insole is larger than the rear foot part. However, the force they impart while walking is relatively similar. Due to the thin and lightweight nature of the three-layer mechanism, it is possible to insert multiple three-layer TENG insoles into a single shoe simultaneously. With these characteristics, we used the three-layer TENG insole to measure the signals generated by the user’s forefoot as Force 1 and their rear foot as Force 2 while walking or running, and calculated their respective forces through the output voltage of the MN-PDMS-TENG. As shown in Figure 10a,c, when the user of the three-layer TENG insole was walking, the voltage measured by the three-layer TENG insole inside the forefoot part was 28 V and the voltage measured by the three-layer TENG insole inside the rear foot part was 26 V. When the user was running, the voltage measured by the three-layer TENG insole under the user’s forefoot was 45 V and the voltage measured by the three-layer TENG insole under user’s rear foot was 15 V, as shown in Figure 10b,d. Through operations with the force sensitivity calibration curve function, F = 12.93 V − 92.10, we could determine the stepping forces on the forefoot and rear foot of this user during walking to be 269.94 N and 244.08 N, respectively, and the stepping force on the forefoot and rear foot of this user during running to be 489.75 N and 101.85 N, respectively.

**Figure 11:**
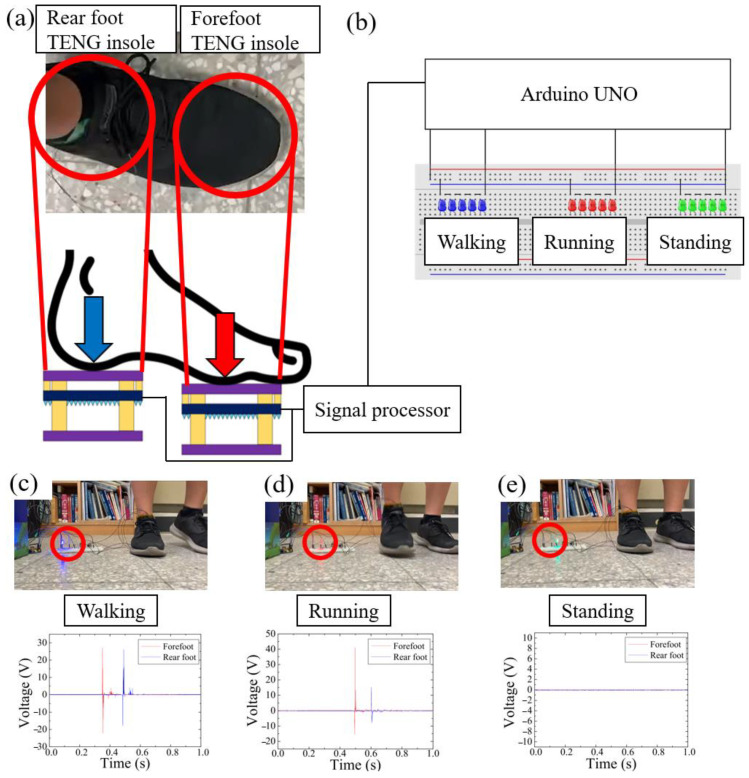
Human stepping state self-powered sensing system with two three-layer TENG insoles as self-powered sensors: (**a**) two insolated three-layer TENG insoles were used as the self-powered sensor to sense the users’ stepping force on the forefoot and rear foot inside the right shoes; (**b**) Arduino UNO was used as the MCU to receive the signal produced by the three-layer TENG insoles, and three groups of LEDs (blue, red, and green) were used to indicate corresponding states; (**c**) when the difference in force between the forefoot and rear foot was less than 300 N, the blue LEDs were on, which signaled walking, as indicated with the red circle; (**d**) when the difference in force between the forefoot and rear foot was higher than 300 N, the red LEDs were on, which signaled running, as indicated with the red circle; (**e**) when the stepping signal had stopped for 2 s, the green LEDs were on, which signaled standing, as indicated with the red circle.

With the three-layer TENG insole, we could determine the force signal of the user’s different parts of the foot without any power support. Combining the three-layer TENG insole with an MCU, a self-powered wearable stepping force sensing system was synthesized. This system triggers actions of a corresponding device according to the state of the user stepping. Thus, we built a self-powered human stepping state sensing system, as shown in Figure 11. Arduino UNO was used as the MCU to receive the signal produced by the three-layer TENG insoles, as shown in Figure 11a,b; process the signals into force signals; and send the corresponding control signal to the corresponding devices. Two separate, insulated three-layer TENG insoles were used as self-powered sensors to detect the user’s stepping force in both the forefoot and rear foot regions, as depicted in Figure 11a. Additionally, three sets of LEDs (blue, red, and green) served as indicators to display the respective states sensed by the three-layer TENG insole, namely, walking, running, and standing, as illustrated in Figure 11b.

In this example, we defined the user as walking when the difference of force between the forefoot and rear foot was less than 300 N and triggers the blue LEDs, as shown in Figure 11c; the user was running when the difference in force between the forefoot and rear foot was larger than 300 N, which triggered the red LEDs, as shown in Figure 11d; and the user was standing when the signal of the PDMS-TENG had stopped for more than 2 s, which triggered the green LEDs, as shown in Figure 11e.

The video showing the human stepping state self-powered sensing system accurately determined the difference in the signal between walking, running, and standing, and transformed the voltage signal from the self-powered sensors into force data and drove corresponding devices depending on the state of the user. This demonstrates the capability of the three-layer TENG insole to be used as a self-powered sensor in the field of sports monitoring and rehabilitation. These devices could be adapted into wearable devices such as smart watches or heart-rate monitors. Compared with the full-time monitor, the human stepping state self-powered sensing system saves a lot of power because it turns the device on and off without any other operation. This is useful for people who need to record their sports status or heart rate many times a day. The system automatically activates and deactivates the heartbeat monitoring device to prevent potential heart injuries resulting from users forgetting to manually activate the device before engaging in exercise.

## 4. Conclusions

In this study, a three-layer TENG insole with a microneedle PDMS dielectric layer and NBCC conductive layer was developed without using any brittle and fragile material, and was utilized as a sensor in a human stepping state self-powered sensing system to sense the user’s state. The flexible and stable microstructure of the PDMS dielectric layer was demolded using a CO_2_ laser, developing a PMMA mold to create the microneedle surface structure and coat on the PET to withstand the shape of the dielectric layer. The microneedle structure increased the electrical output profile of MN-PDMS-TENG: the Voc values of the PDMS-TENG with and without the microneedle was 129.2 V and 54.6 V, respectively, which is an increase of 237%. The Isc values were 64.00 μA and 26.16 μA, respectively, which is an increase of 245%. The power values were 4.1 mW and 684 μW, respectively, which is an increase of 599%. For charging the 1 μF capacitor, the PDMS-TENG took 4 s to reach 1.21 V and the MN-PDMS-TENG took 3 s to reach 1.75 V. In terms of lighting the LEDs, the MN-PDMS-TENG lit up to 120 LEDs and the PDMS-TENG lit up to 90 LEDs. The NBCC conductive layer was constructed using a combination of conductive cloth and a nylon base. This design aimed to ensuring the insole’s durability under human stepping while maintaining comfort. The three-layer TENG insole mechanism was structured as the NBCC, MN-PDMS-TENG, the nylon support layer, and the PE spacers, to keep the best stroke of the MN-PDMS-TENG at 6 mm and ensure that every step achieved a complete contact and separation movement. There was no hard and brittle material included in the mechanism, which kept the insole soft and stretchable. The three-layer TENG insole output a Voc of 87.2 V and lit up 13 LEDs. With the data of voltage and force under different forces, the force sensitivity of the three-layer TENG insole was 0.07734 V/N with the coefficient of determination R^2^ = 0.91 under human stepping. The function between force and Voc was derived as F = 12.93 V − 92.10. The self-powered human stepping state sensing system was established using two three-layer TENG insoles, positioned beneath the user’s forefoot and rear foot. An Arduino UNO served as the microcontroller (MCU), and three groups of LEDs functioned as display devices. Calculating the difference in force data between the forefoot and rear foot with the Arduino UNO, the user can control the devices performing the corresponding operations with different stepping states, including walking, running, and standing. This study demonstrates the potential of the three-layer TENG insole to be used as a self-powered sensor and its application in sports monitoring and rehabilitation.

## Figures and Tables

**Table 1:** Comparison of TENG’s electrical output performance between other fiber-based TENGs.

Material	Voc	Isc	Power	LEDs	Ref.
LMPA-cotton	85.4 V	2.32 μA	348.5 μW/m	30	[27]
Cu-CNT-Silicone	140 V	7.5 µA m^–1^	5.5 µW	X	[28]
CA-PES-PS+C-PS	115.2 V	9.21 μA,	X	60	[29]
H_3_PO_4_/PVA-silicone-carbon fiber-silicone	42.9 V	0.51 μA	∼1.12 μW	1	[11]
**PET-MN-PDMS-Al**	**73.6 V**	**36.00 μA**	**1.296 mW** **0.264 W/m^2^**	**90**	Previous work [25]
**PET-MN-PDMS-Al**	**129.2 V**	**64.00 μA**	**4.10 mW** **1.14 W/m^2^**	**120**	**This research**

**Table 2:** Comparison of application between other insole TENGs.

Material	Mechanism	Sensing Range (N)	Ref.
Teflon-NR-ITO	Two layers	2~10 N	[11]
Nylon-F-PODN	Two layers	1~15 N	[30]
Rubber-Cu	Two layers	5~40 N	[31]
**PET-PDMS-Al**	**Three-layer insole mechanism**	**300~550 N** **(~human weight)**	**This research**

## Data Availability

Data are the coauthors’ research results and schematic drawings.
